# Migrant integration policies and health inequalities in Europe

**DOI:** 10.1186/s12889-016-3095-9

**Published:** 2016-06-01

**Authors:** Margherita Giannoni, Luisa Franzini, Giuliano Masiero

**Affiliations:** 1 Institute of Economics, Università della Svizzera Italiana, via Buffi 13, Lugano, 6900 Switzerland; 2 Department of Economics, Università degli Studi di Perugia, via Pascoli 20, Perugia, 06123 Italy; 3 School of Public Health, University of Maryland, College Park, MD, 20742 USA; 4 Department of Management, Information and Production Engineering, University of Bergamo, Via Pasubio 7b, Dalmine (BG), 24044 Italy

**Keywords:** Health inequalities, Socio-economic determinants of health, Migrant integration policy, Migration and health in Europe

## Abstract

**Background:**

Research on socio-economic determinants of migrant health inequalities has produced a large body of evidence. There is lack of evidence on the influence of structural factors on lives of fragile groups, frequently exposed to health inequalities. The role of poor socio-economic status and country level structural factors, such as migrant integration policies, in explaining migrant health inequalities is unclear. The objective of this paper is to examine the role of migrant socio-economic status and the impact of migrant integration policies on health inequalities during the recent economic crisis in Europe.

**Methods:**

Using the 2012 wave of Eurostat EU-SILC data for a set of 23 European countries, we estimate multilevel mixed-effects ordered logit models for self-assessed poor health (SAH) and self-reported limiting long-standing illnesses (LLS), and multilevel mixed-effects logit models for self-reported chronic illness (SC). We estimate two-level models with individuals nested within countries, allowing for both individual socio-economic determinants of health and country-level characteristics (healthy life years expectancy, proportion of health care expenditure over the GDP, and problems in migrant integration policies, derived from the Migrant Integration Policy Index (MIPEX).

**Results:**

Being a non-European citizen or born outside Europe does not increase the odds of reporting poor health conditions, in accordance with the “healthy migrant effect”. However, the country context in terms of problems in migrant integration policies influences negatively all of the three measures of health (self-reported health status, limiting long-standing illnesses, and self-reported chronic illness) in foreign people living in European countries, and partially offsets the “healthy migrant effect”.

**Conclusions:**

Policies for migrant integration can reduce migrant health disparities.

**Electronic supplementary material:**

The online version of this article (doi:10.1186/s12889-016-3095-9) contains supplementary material, which is available to authorized users.

## Background

Achieving health equity through the reduction of health inequalities has been included among the measures of health systems performance by the World Health Organization [1]. Research on socio-economic determinants of health inequalities in general, and on migrants health inequalities in particular, has produced a large body of evidence, mainly for the US and Europe [1, 2]. In Europe, there is substantial evidence on socio-economic inequalities in health, starting from Whitehall studies in the 1980s [3]. At the same time, given the persistence of socio-economic health inequalities [4], the European Union has encouraged action in many countries providing a framework and the principles to tackle health inequalities [5–7]. On the one hand, the importance of policies aiming at improving opportunities for full social participation, which is considered a key factor for good health, has been the focus of many documents at all institutional levels [8–10]. On the other hand, like in the US [3], there is lacking evidence on how structural factors, such as migrant integration policies, influence the lives of fragile groups, such as migrants who are generally affected by socio-economic health inequalities [11]. A recent European survey shows that migrants suffer from health inequalities, despite the fact that they are often healthier than natives, which is described in the literature as the “healthy migrant effect” [12-13]. Moreover, despite the fact that migration is increasingly recognized as an independent social determinant of health [11], poorer socio-economic conditions could derive from social exclusion mechanisms that characterize the migrant status and ethnic origin [14]. Other studies report that migrants living in countries with poor integration policies experience poorer socio-economic and health outcomes, but do not estimate the effects of the socio-political context of migrants integration on health [15]. Therefore, further evidence is needed in order to better address the development of interventions to promote the healthy integration of migrants into the European society. Moreover, it seems important to investigate with recent data whether the migrant status can be considered an autonomous and significant determinant of health inequalities in Europe (EU), after controlling for other socio-economic determinants, such as income and education.

The analysis of cross sectional data from the Eurostat EU-SILC dataset for a set of 14 European countries before the recent economic crisis (year 2007) shows that being a non-EU citizen and living in the EU is not a significant determinant of self-assessed health inequalities “per se” [16]. What matters instead is the fact of living in a country with problems in migrant integration. The Migrant Integration Policy Index (MIPEX) has been recently updated. Therefore, it is now possible to test if migrant integration policies influenced health inequalities in the EU during the economic recession occurred after 2009, which was associated with worsening health inequalities in several countries, e.g. Greece [17].

Using the 2012 wave of Eurostat EU-SILC data for a set of 23 European countries, we estimate multilevel logit and ordered logit models for self-assessed poor health (SAH), self-reported limiting long-standing illnesses (LLS) and self-reported chronic illness (SC). We estimate two-level models with individuals nested within countries, allowing for both individual socio-economic determinants of health and country-level characteristics (healthy life years expectancy, proportion of health care expenditure over the GDP, and the number of problems in migrant integration policies, derived from the Migrant Integration Policy Index). We complement the global analysis based on all countries, with a two-steps analysis at country level.

In the Methods section we present the conceptual model and the empirical approach. Data are described in the Data section. The presentation of the results and discussion will follow. Finally, in the last section we briefly conclude.

## Methods

**Fig. 1:**
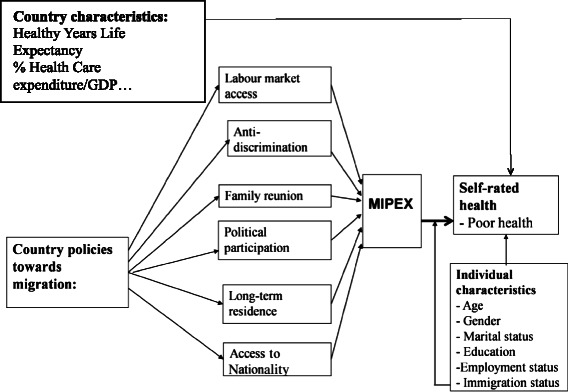
The conceptual model. Source: adapted from Franzini and Giannoni [20]

The conceptual model for the first step of the analysis is drawn from previous studies [16] (see Fig. 1). The theoretical framework is based on socio-ecological models assuming that self-assessed health is affected by a large set of determinants at multiple levels. The most important determinants are socio-economic factors, social and physical environments, healthcare use, and health behaviors [18]. Being a non-European citizen or non-born in Europe, as a proxy for migrant status, is considered one of the socio-economic determinants of health acting at the individual or family level [19]. At the group level, socio-economic factors contribute to unequal social and physical environmental exposures, which increase health inequalities [20]. In this context, the aim is to test if migrant policies affect the socio-economic environment in which both migrants and non-migrants live. If individuals live in a country where there are problems in terms of granting rights to migrants, this could reasonably negatively affect the way they live and, ultimately, their health. This hypothesis is tested in the present analysis by considering country policies towards migration as a component of the social environment in which both migrants and non-migrants live. Therefore, migrant policies are introduced at the country level using a migrant integration policy variable in order to explain the observed socio-economic inequalities in health. Migrant integration policies at country level may influence health through several pathways. They are part of the social context of the country where individuals live, and as such they can affect the health of all people living in the country. Furthermore, their specific interaction with the status of non-EU citizenship, can affect migrants health status at the individual level, such as other individual socio-economic determinants (e.g. income, occupation, education, etc.).

We use multilevel models with a dataset of individual observations made available by Eurostat through the release of the 2012 wave of EU-SILC cross-sectional data [21]. Using multilevel models allows to estimate the proportion of the variation in health that can be explained by the social status, controlling for other determinants of health at both individual and country level, as well as country level unobserved factors [16]. Moreover, by using multilevel models it is possible to introduce simultaneously individual level variables and country level factors, such as country specific policies and attitudes towards migration. The use of cross-sectional data has its own limitations, partially overcome by multilevel techniques. In this case, we decided not to use the longitudinal survey. The main reason is that information on the citizenship status or country of birth is limited compared to cross sectional waves, and it is not always representative at country level. Moreover, cross-sectional data are overall richer in terms of information recorded, i.e. more variables are available in cross sectional waves than in the longitudinal version of the EU-SILC dataset [22]. For each response variable, we carried out two analyses: a global analysis and a two-step analysis. The global analysis involves the entire study sample, whereas the two-step analysis is conducted by running separate regressions for each country using only individual level variables. Both analyses treat self-reported measures of health status as dependent variables.

In the global analysis, due to the multistage sampling design used to collect the data and considering the nature of the response variables, we use two-level models with individuals nested within countries. In the first step of the analysis, multilevel ordered mixed effects logit models are estimated for the dependent variables: self-assessed poor health and self-reported limiting severe or very severe long standing illnesses. These models allow for the estimation of the direct effect of individual-level and group-level explanatory variables, as well as interactions between levels [23].

We consider the following two-level mixed effects ordered logistic model for the dependent variable, *y*_ij_ (for individual *i*, country *j*). The probability of observing outcome *k* for response *y*_*ij*_ is:

1 $$ \begin{array}{c}{p}_{ij}= Pr\left({y}_{ij}=k\Big|\kappa, {u}_j\right)= Pr\left({\kappa}_{k-1}<{\eta}_{ij}+{\epsilon}_{it}\le {\kappa}_k\right)\\ {}=\frac{1}{1+ exp\left(-{\kappa}_k+{\eta}_{ij}\right)}-\frac{1}{1+ exp\left(-{\kappa}_{k-1}+{\eta}_{ij}\right)}\end{array} $$

where

*η*_*ij*_ *= X*_*ij*_ *β* + *Z*_*ij*_ *u*_*j*_ + *offsett*_*ij*_, k_0_ is taken as -∞, and k_k_ is taken as + ∞. X_*ij*_ are the demographic and socio-economic explanatory variables at individual level (level 1), and Z_*j*_ are the explanatory variables at country level (level 2). *X*_*ij*_ does not contain a constant term because its effect is absorbed into the cutpoints.

For cluster (country) *j, j = 1,…, M* (with cluster j consisting of *i = 1,…,n*_*j*_ observations), the conditional distribution of *y*_*j*_ *= (y*_*j1*_ *,…, y*_*jnj*_ *)’* given a set of cluster-level random effects *u*_*j*_ is

2 $$ f\left({y}_j\Big|\kappa, {u}_j\right)={\displaystyle {\prod}_{i=1}^{n_j}{p}_{ij}^{I_k\left({y}_{ij}\right)}}= exp{\displaystyle {\sum}_{i=1}^{\eta_j}\left\{{I}_k\left({y}_{ij}\right) log{p}_{ij}\right\}} $$

where

3 $$ {I}_k\left({y}_{ij}\right)=\left\{\begin{array}{c}\hfill 1\kern1.25em \mathrm{if}\ {y}_{ij}=k\hfill \\ {}\hfill 0\kern0.75em \mathrm{otherwise}\hfill \end{array}\right. $$

Moreover, we estimate multilevel mixed-effects logistic regression models for self-reported chronic illness. In order to analyze the differential influence of individual characteristics over health, further models are estimated adding the interactions between the ecological variables and the individual characteristics. In particular, we check if problems in policies for migrant integration at country level influence non-European born or non-European citizens’ health differently than local citizens’ health. Moreover, in order to take into account possible interaction effects between socio-economic and demographic conditions and the migrant status, the key variable “Non-EU citizen or born outside the EU” is interacted with individual socio-economic characteristics.

### Data

The first part of the analysis is based on cross-sectional micro-data from the Eurostat, EU-SILC, reference year: cross sectional 2012 [21]. Participants are adults regularly residents in European countries. We select countries for which citizenship status and country of birth is recorded and the sample is representative of the population.^1^ The final sample has 375,110 observations grouped in 23 countries. Table 1 shows the summary statistics for individual and country variables used in the analysis. The three dependent variables modeled are: self-assessed poor health, self-reported limiting long-standing illnesses and self-reported chronic illness. Self-assessed health is measured by the answer to the question “*How is your health in general? Is it …*”. Respondents choose from a scale of five options: very good, good, fair, bad and very bad. SAH is one of the most widely used indicators of health in survey research, and recommended by both the World Health Organization and the European Union Commission. Evidence shows that SAH is a strong and independent predictor of morbidity and mortality, as there is an association between SAH and mortality even after adjusting for prevalent diseases and health behavioral factors [24]. Therefore, the analysis looks at the risk factors of SAH taking into account the ordered nature of the variable. Estimates are reported for ordered logit models. To complement the analysis, we also considered other measures of health: limiting long-standing illness and chronic diseases. Limiting long standing illness is measured by the answer to the question: “*For at least the past 6 months, to what extent have you been limited because of a health problem in activities people usually do? Would you say you have been …* “. Respondents choose their answer among the following three options: severely limited, limited but not severely, not limited at all. For the purpose of this study, we consider the ordered nature of the variable and estimate ordered logit models. Chronic illness is measured by the answer to the question:”*Do you have any longstanding illness or [longstanding] health problem?”.* In this case, the estimates are reported for logit models. Moreover, in order to perform the two-step analysis, responses for each of the three measures of health are condensed into a dichotomous variable.

**Table 1:** Summary statistics and variables definition

Description	Data source	Mean	Std. Dev.	Min	Max
Individual level:					
*Gender: =1 if male, 0 otherwise*	EU-SILC 2012 C.S. wave	0.50	0.50	0	1
*Age*	EU-SILC 2012 C.S. wave	48.50	18.14	16	80
*Age squared*	EU-SILC 2012 C.S. wave	2684	1779	256	6400
*Low education: =1 if highest ISCED level up to secondary lower education level, 0 otherwise*	EU-SILC 2012 C.S. wave	0.33	0.47	0	1
*Unemployed: =1 if unemployed, 0 otherwise*	EU-SILC 2012 C.S. wave	0.07	0.25	0	1
*Student: =1 if student, 0 otherwise*	EU-SILC 2012 C.S. wave	0.08	0.27	0	1
*Retired or Unable to work: =1 if retired or unable to work, 0 otherwise*	EU-SILC 2012 C.S. wave	0.28	0.45	0	1
*Housework: =1 if housework, 0 otherwise*	EU-SILC 2012 C.S. wave	0.07	0.25	0	1
*Self-employee: =1 if self-employed, 0 otherwise*	EU-SILC 2012 C.S. wave	0.07	0.25	0	1
*Marital status: =1 if not married; 0 otherwise*	EU-SILC 2012 C.S. wave	0.28	0.45	0	1
*Widow: =1 if widowed, 0 otherwise*	EU-SILC 2012 C.S. wave	0.08	0.27	0	1
*Separated or divorced: =1 if separated or divorced, 0 otherwise*	EU-SILC 2012 C.S. wave	0.07	0.27	0	1
*Foreign non-EU citizen or non-EU born: =1 if citizen of a non-EU country or was born in a non-EU country, 0 otherwise*	EU-SILC 2012 C.S. wave	0.06	0.23	0	1
*Log of individual income (equivalised with OECD scale)*	EU-SILC 2012 C.S. wave	9.35	1.15	0	14.61
Country level (n. countries =23):					
*% Health care expenditure on GDP*	Eurostat Statistics ^a^	9.09	1.97	5.11	12.43
*Healthy life years*	Eurostat Statistics ^b^	61.67	4.23	53.25	72.1
*N. of problematic areas of integration policy (measured by MIPEX 2010 data)*	MIPEX data ^c^	1.98	1.39	0	5
Interaction term: (N. of problematic areas of integration policy * foreign non-EU citizen or non-EU born)		0.14	0.69	0	5

^a^Available at http://ec.europa.eu/eurostat/data/database, last accessed 18th August 2014

^b^The indicator of healthy life years (HLYs) measures the number of remaining years that a person of specific age is expected to live without any severe or moderate health problem. The notion of health problem for Eurostat’s HLY is reflecting a disability dimension and is based on self-perception. This aims to measure the extent of any limitation, for at least six months, because of health problems that may have affected respondents regarding activities they usually do (the so-called GALI - Global Activity Limitation Instrument foreseen in the annual EU-SILC survey). The indicator is therefore also called disability-free life expectancy (DFLE). HLY is a composite indicator that combines mortality data with health status data

^c^Available at http://www.mipex.eu, last accessed 18th August 2014

Table 2 shows country-level statistics for the total sample of observations used and for the dependent variables. There is a noticeable variation across countries in all the three health measures. The percentage of individuals with poor or very poor self-assessed health shows the largest variation, from a minimum of 3 % (Malta, Switzerland) to a maximum of 25 % (Croatia). Conversely, the variation in the percentage of people with severe or very severe limitations in daily life is less remarkable, and ranges between 10 % (Malta) and 37 % (Finland and Portugal). Finally, the proportion of people with at least one chronic disease is the lowest in Bulgaria (18 %) and the highest in Finland (50 %). Overall, we do not observe a clear geographical gradient (North–south or East–west).

**Table 2:** Sample statistics for the dependent variables^a^

Country	% in sample	% Non-EU citizens & non-EU born	% SAH- self-assessed health (ordered)						% Limitations in daily life (ordered)				% at least 1 chronic disease
			% Poor or very poor SAH	Very good	Good	Fair	Bad	Very bad	Severe/very severe limitations	No limitations	Yes, limited	Yes, strongly limited	
AT	2	12	9	34	36	21	7	2	28	73	18	10	33
BG	2	0	12	18	49	21	9	3	18	82	14	4	18
CH	2	11	3	33	49	15	3	1	19	81	13	6	34
DE	18	16	9	18	47	26	7	2	34	66	23	11	36
DK	1	4	8	16	27	14	4	2	29	71	21	8	31
EE	0	22	16	7	35	25	11	2	33	68	23	10	44
EL	2	8	9	47	28	16	7	3	23	77	13	10	23
ES	10	10	7	22	52	18	6	2	22	78	17	5	24
FI	1	2	8	9	27	16	4	1	37	63	29	8	50
FR	13	7	8	25	43	23	7	1	25	75	16	9	36
HR	1	10	25	8	18	15	12	3	23	77	18	5	29
HU	2	0	16	16	41	26	12	4	25	75	17	8	36
IT	14	6	12	13	53	19	9	3	29	71	20	9	23
LT	1	6	20	6	31	29	14	3	26	74	18	8	29
LU	0	11	7	24	49	19	6	2	20	81	14	6	20
LV	0	20	15	4	42	38	12	3	29	71	22	7	36
MT	0	4	3	19	55	23	3	0	10	90	7	3	29
NL	4	10	7	13	28	11	3	0	31	69	24	7	37
PT	2	5	14	8	40	34	13	5	37	63	15	22	33
RO	5	0	9	28	42	20	8	2	26	74	18	8	19
SE	2	8	5	19	26	9	2	1	18	83	11	7	36
SK	1	0	12	21	44	22	10	3	33	67	23	10	30
UK	13	11	8	38	36	17	6	2	22	78	11	11	32
TOTAL	100	9	10	19	39	21	8	2	26	73	18	9	31

^a^Percentages obtained by using individually weighted data

Data source: Eurostat - EU-SILC cross sectional Reference year: 2012 [21]

Legend: *AT* Austria, *BG* Bulgaria, *CH* Switzerland, *DE* Germany, *DK* Denmark, *EE* Estonia, *EL* Greece, *ES* Spain, *FI* Finland, *FR* France, *HR* Croatia, *HU* Hungary, *IT* Italy, *LT* Lithuania, *LU* Luxembourg, *LV* Latvia, *MT* Malta, *NL* The Netherlands, *PT* Portugal, *RO* Romania, *SE* Sweden, *SK* Slovak Republic, *UK* United Kingdom

The individual independent variables correspond to socio-demographic (age, sex, marital status and nationality) and socio-economic (educational level, personal income and employment status) dimensions. Education is measured as the highest ISCED level attained. The variable for low education is a dummy variable that takes the value of one if the individual attained up to a lower secondary level of education, and zero otherwise. There is remarkable variation between and within countries in the average level of education attained by non-EU citizens/non-EU born individuals. Southern countries, like Portugal, France, Luxembourg, Italy and Spain, show a higher proportion of low-educated non-EU citizens/non-EU born individuals, as compared to the UK, Finland, Sweden, and most Eastern European countries. The reference individual is a local citizen or EU born living in a country without problems in migrant integration policies.

In order to measure migrant integration policies in European countries we use MIPEX data for 2010, the latest available year of the survey [25]. Today, the MIPEX project is led by CIDOB and the Migration Policy Group, and includes up to 37 national-level organizations, such as think-tanks, NGOs, foundations, universities, research institutes and equality bodies. Research activities are coordinated by the Migration Policy Group, in cooperation with the research partners. Our MIPEX data cover the following six policy areas: labor market mobility, family reunion for third countries nationals, political rights, long-term residence, access to nationality, anti-discrimination policies. MIPEX indicators are on a 0–100 % scale for each policy area, where 100 % is the top score.

**Fig. 2:**
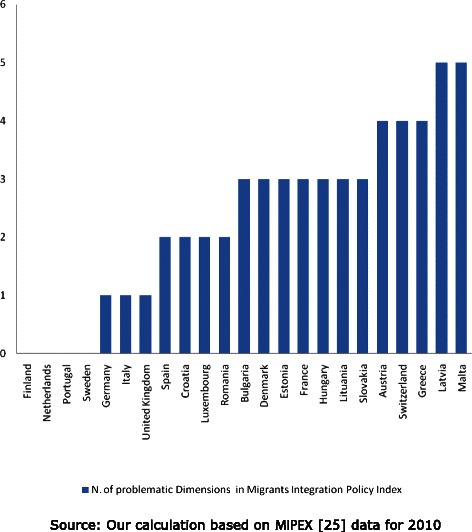
N. of problematic areas in migrant integration policy by country

In order to build a composite measure of migrant policies, we develop an index based on MIPEX data. The index measures the number of problematic policy areas in 2010, i.e. areas ranked with a value below 50 % of the maximum MIPEX score. The problematic migrant policy scale can take values from 0 to 5. For example, in countries scoring the maximum value of the index, such as Latvia, political participation and anti-discrimination policies are limited, while access to citizenship is difficult, labor mobility and access policies are limited. Moreover, procedures for family reunion and long-term residence acquisition are complicated, as well as rights of access to health care. Table 3 shows the distribution of MIPEX scores by area of integration and country. We observe high variation across countries for all 6 areas as well as for the overall score. There is a remarkable correlation between the scores of different dimensions. The overall score more than doubles when moving from countries with problematic integration policies (minimum of 33 in Latvia) to countries with good levels of migrant integration (maximum of 84 in Sweden). The number of problematic dimensions reflects well the overall MIPEX score. We initially tested several alternative specification of the MIPEX index. The sub-dimensions were aggregated using a factor analysis. We also considered all sub-dimensions separately as independent variables in the model. However, the best and most parsimonious specification was obtained by using the number of problematic dimensions. This approach is also particularly useful for the interpretation of the results. According to the index, countries such as Finland, The Netherlands, Portugal, Finland and Sweden appear to be less problematic than Latvia, Malta, Greece, Switzerland and Estonia (Fig. 2).

**Table 3:** MIPEX data

Country	MIPEX indicators - 2010							
	MIPEX dimensions						Number of problematic dimensions^a^	Overall score^b^
	Anti discrimination	Access to nationality	Political participation	Long term residence	Family reunion	Labour market		
AT	40	22	33	58	41	56	4	40
BG	80	24	17	57	51	40	3	45
CH	31	36	59	41	40	53	4	43
DE	48	59	64	50	60	77	1	60
DK	47	33	62	66	37	73	3	53
EE	32	16	28	67	65	65	3	45
EL	50	57	40	56	49	49	4	50
ES	49	39	56	78	85	84	2	65
FI	78	57	87	58	70	71	0	70
FR	77	59	44	46	52	49	3	54
HR	58	29	17	67	56	55	2	47
HU	75	31	33	60	61	41	3	50
IT	62	63	50	66	74	69	1	64
LT	55	20	25	57	59	46	3	44
LU	48	66	78	56	67	48	2	62
LV	25	15	18	59	46	36	5	33
MT	36	26	25	64	48	43	5	40
NL	68	66	79	68	58	85	0	71
PT	84	82	70	69	91	94	0	81
RO	73	29	8	54	65	68	2	49
SE	88	79	75	78	84	100	0	84
SK	59	27	21	50	53	21	3	38
UK	86	59	53	31	54	55	1	56

Data source: MIPEX (Migrant Integration Policy Index) [25]

^a^Problematic dimensions are defined as scoring <50

^b^Overall score not including Education

Legend: *AT* Austria, *BG* Bulgaria, *CH* Switzerland, *DE* Germany, *DK* Denmark, *EE* Estonia, *EL* Greece, *ES* Spain, *FI* Finland, *FR* France, *HR* Croatia, *HU* Hungary, *IT* Italy, *LT* Lithuania, *LU* Luxembourg, *LV* Latvia, *MT* Malta, *NL* The Netherlands, *PT* Portugal, *RO* Romania, *SE* Sweden, *SK* Slovak Republic, *UK* United Kingdom

In the estimation, we included country-level variables controlling for both the health care system and the overall economy. The following country-level variables were obtained from the OECD Health Data and the Eurostat statistics [26, 27]: the *Gini* index for income inequality, poverty, pollution and homicide rates, the number of hospital beds per 1000 inhabitants, the proportion of immigrants amongst residents, the Gross Domestic Product (GDP) per capita, total healthcare expenditure as a share of GDP, the healthy life years expectancy, and the level of corruption. Out of these variables, only two were significant in some models, namely: the healthy years life expectancy and the healthcare expenditure as a share of GDP. Therefore, the results reported were obtained by controlling for these variables.

## Results and discussion

Results from the estimation of multilevel ordered logit models for self-assed health status and for the probability of reporting limitations in daily life are reported in Tables 4 and 5, respectively. Table 6 shows the results for multilevel logit models for the probability of reporting chronic conditions. For each dependent variable we estimated 6 models. Model 1 includes individual demographic and socio-economic determinants. Model 2 adds the country level characteristics, healthy life years expectancy and the proportion of health care expenditure over the GDP. Model 3 adds the country level variable measuring problems in migrant integration policies. Conversely, Model 4 adds an interaction term between the non-EU citizenship or born status and the policy variable measuring the country-level number of problematic migrant policy areas. Model 5 adds both the policy variable and the interaction term with the variable measuring non-EU migrant status. Finally, Model 6 adds interactions between being a non-EU citizen or born and socio-economic (SES) factors.^2^ The interaction term between the policy and migrant status allows for the estimation of the marginal impact of integration policies on non EU-migrants.

**Table 4:** Multilevel ordered logit estimates - probability of reporting poor/very poor/fair/good/very good health– Year: 2012^a^

Variables	Model 1	Model 2	Model 3	Model 4	Model 5	Model 6
Individual (level 1)						
*Non-EU migrant (citizen or born outside the EU)*	1.135***	1.166***	1.114***	1.064*	1.073**	0.988
Country (level 2)						
*Health care expenditure on GDP*		0.882***	0.928***	0.892***	0.894***	0.871***
*Healthy life expectancy*		0.929***	0.942***	0.948***	0.946***	0.946***
*N. of problems with migrant integration policies*			1.010***		1.040***	1.010***
N. of problems with migrant integration policies * non-EU migrant				1.035***	1.025**	1.038***
Interactions: non-EU migrant (citizen or born outside the EU) * individual-level variables						
Non-EU migrants * low education						1.225***
Non-EU migrants * low income						1.068*
Non-EU migrant * unemployed						0.845***
Non-EU migrant * housework						0.943
Non-EU migrant * self employed						1.028
Non-EU migrant * not married						1.026
Non-EU migrant * divorced						1.005
Non-EU migrant * widow						0.756*
*Cut1*	1.082	0.006***	0.018***	0.029***	0.021***	0.001***
*Cut2*	16.157***	0.085***	0.272***	0.445***	0.317***	0.015***
*Cut3*	107.077***	0.568***	1.811***	2.954***	2.108***	0.095***
*Cut4*	688.125***	3.641***	11.663***	18.998***	13.566***	0.588***
*Sigma2 u*	1.084***	1.359***	1.085***	1.176***	1.115***	1.090***
*Chi2*	98000	110000	110000	110000	110000	98000
*N. of countries*	23 (AT BG CH DE DK EE EL ES FI FR HR HU IT LT LU LV MT NL PT RO SE SK)					
*N. of observations*	332011 (all models)					

Legend: * *p* < 0.05; ** *p* < 0.01; *** *p* < 0.001

*AT* Austria, *BG* Bulgaria, *CH* Switzerland, *DE* Germany, *DK* Denmark, *EE* Estonia, *EL* Greece, *ES* Spain, *FI* Finland, *FR* France, *HR* Croatia, *HU* Hungary, *IT* Italy, *LT* Lithuania, *LU* Luxembourg, *LV* Latvia, *MT* Malta, *NL* The Netherlands, *PT* Portugal, *RO* Romania, *SE* Sweden, *SK* Slovak Republic, *UK* United Kingdom

^**a**^Odds ratios. Estimates obtained by controlling for individuals age, gender, education, individual income, occupational status, marital status

Source: our calculation based on Eurostat [21, 27], OECD [26] data for 2012 and on MIPEX [25] data

**Table 5:** Multilevel ordered logit estimates – dependent variable: probability of reporting severe/very severe/no limitations in daily life - Year: 2012^a^

Variables	Model 1	Model 2	Model 3	Model 4	Model 5	Model 6
Individual (level 1)						
*Non-EU migrant (citizen or born outside the EU)*	1.034*	1.025	1.027	0.966	0.942	0.894**
Country (level 2)						
*Health care expenditure on GDP*		1.094***	1.096***	1.078***	0.928***	1.067***
*Healthy life expectancy*		0.920***	0.919***	0.909***	0.926***	0.927***
*N. of problems with migrant integration policies*			0.988***		0.932***	0.972***
N. of problems with migrant integration policies * non-EU migrant				1.030**	1.040***	1.046***
Interactions: non-EU migrant (citizen or born outside the EU) * individual-level variables						
Non-EU migrants * low education						1.262***
Non-EU migrants * low income						1.068
Non-EU migrant * unemployed						0.844**
Non-EU migrant * housework						0.884
Non-EU migrant * self employed						1.037
Non-EU migrant * not married						0.852**
Non-EU migrant * divorced						1.006
Non-EU migrant * widow						0.905
*Cut1*	15.564***	0.258***	0.235***	0.114***	0.106***	0.026***
*Cut2*	80.927***	1.346***	1.224***	0.593***	0.554***	0.131***
*Sigma2 u*	1.271***	1.053***	1.064***	1.165***	1.032***	1.213***
*Chi2*	56000	57000	60000	58000	58000	52000
*N. of countries*	23 (AT BG CH DE DK EE EL ES FI FR HR HU IT LT LU LV MT NL PT RO SE SK)					
*N. of observations*	340920 (all models)					

Legend: * *p* < 0.05; ** *p* < 0.01; *** *p* < 0.001

*AT* Austria, *BG* Bulgaria, *CH* Switzerland, *DE* Germany, *DK* Denmark, *EE* Estonia, *EL* Greece, *ES* Spain, *FI* Finland, *FR* France, *HR* Croatia, *HU* Hungary, *IT* Italy, *LT* Lithuania, *LU* Luxembourg, *LV* Latvia, *MT* Malta, *NL* The Netherlands, *PT* Portugal, *RO* Romania, *SE* Sweden, *SK* Slovak Republic, *UK* United Kingdom

^**a**^Odds ratios. Estimates obtained by controlling for individuals age, gender, education, individual income, occupational status, marital status

Source: our calculation based on Eurostat [21, 27], OECD [26] data for 2012 and on MIPEX [25] data

**Table 6:** Multilevel logit estimates for the probability of reporting chronic diseases –Year: 2012^a^

Variables	Model 1	Model 2	Model 3	Model 4	Model 5	Model 6
Individual (level 1)						
*Non-EU migrant (citizen or born outside the EU)*	1.002	1.002	1.002	0.879***	0.879***	0.843***
Country (level 2)						
*Health care expenditure on GDP*		1.084***	1.088***	1.086***	1.089***	1.090**
*Healthy life expectancy*		0.966***	0.966***	0.966***	0.966***	0.966*
*N. of problems with migrant integration policies*			1.024		1.020	1.006
N. of problems with migrant integration policies * non-EU migrant				1.052***	1.052***	1.043***
Interactions: non-EU migrant (citizen or born outside the EU) * individual-level variables						
Non-EU migrants * low education						1.111**
Non-EU migrants * low income						1.127**
Non-EU migrant * unemployed						0.849**
Non-EU migrant * housework						0.821**
Non-EU migrant * self employed						0.97
Non-EU migrant * not married						0.944
Non-EU migrant * divorced						1.083
Non-EU migrant * widow						1.155*
*Constant*	0.478***	0.178***	0.16952	0.174	0.167	0.126***
*Sigma u*	0.556***	0.371***	0.377***	0.376***	0.375***	0.354***
*Rho*	0.051***	0.041***	0.041***	0.041***	0.041***	0.037***
*Chi2*	51048	51051	51051	51066	51066	47000
*N. of countries*	23 (AT BG CH DE DK EE EL ES FI FR HR HU IT LT LU LV MT NL PT RO SE SK)					
*N. of observations*	340524 (all models)					

Legend: * *p* < 0.05; ** *p* < 0.01; *** *p* < 0.001

*AT* Austria, *BG* Bulgaria, *CH* Switzerland, *DE* Germany, *DK* Denmark, *EE* Estonia, *EL* Greece, *ES* Spain, *FI* Finland, *FR* France, *HR* Croatia, *HU* Hungary, *IT* Italy, *LT* Lithuania, *LU* Luxembourg, *LV* Latvia, *MT* Malta, *NL* The Netherlands, *PT* Portugal, *RO* Romania, *SE* Sweden, *SK* Slovak Republic, *UK* United Kingdom

^a^Odds Ratios. Estimates obtained by controlling for individuals age, gender, education, individual income, occupational status, marital status

Source: our calculation based on Eurostat [21, 27], OECD [26] data for 2012 and on MIPEX [25] data

For all the three measures of the health status, the probability of reporting poor health is affected by socio-economic determinants, as it is suggested by the empirical literature. The odds of reporting poor health increase with age, and decrease with education, income, employment status, and widow, separated, divorced or single status. Working individuals, either as employee or self-employed, report better health as compared to non-working individuals. In order to focus on the main variables of interest, the coefficients of individual demographic and SES characteristics are not reported in the tables. All these variables are statistically significant at 1 % level in all models.

Looking at the results for SAH, Model 1 shows that being a non-EU citizen or born outside the EU affects positively the probability of reporting poor health (Table 4). Model 2 adds the country level characteristics: healthy years life expectancy and the proportion of total health care expenditure over the GDP. Both these variables seem to exert a protective effect on health. Living in a country with higher healthy years life expectancy and proportion of total health expenditure on GDP decreases the odds of reporting poor health. In Model 3, the country level variable measuring problems in migrant integration policies appears to increase the odds of reporting poor health. Model 4 shows that the negative effect on health of being a non-EU citizen is mediated by the fact of living in countries where the acquisition of nationality, political rights, long-term residence, labor market mobility, family reunion and anti-discrimination policies are unfavorable to migrants. The results of Model 5 show that both the policy variable and its interaction with the non-EU migrant status continue to be significant. Therefore, in both Model 4 and Model 5 being a non-EU migrant and living in countries where there are problems in terms of integration policies increases the odds of reporting poor health. Moreover, Model 5 suggests that the health status of the non-EU migrant is affected more strongly than the health status of the baseline individual as the number of problems in integration policies increases. Adding the interaction terms between the migrant status and SES variables (Model 6) does not significantly change the estimated odd of the migrant integration policy variable, and its interaction with the non-EU migrant status as compared to the other models. However, the non-EU migrant status appears to be associated with lower odds of reporting poor health, although not significantly. It follows that the coefficient of the non-EU migrant status may unveil a possible “healthy migrant effect”. Moreover, low levels of education and income tend to decrease the health condition of non-EU migrants, as suggested by the interaction terms between SES and the non-EU migrant status. The interaction term between the non-EU migrant status and the policy variable might be interpreted as a measure of inequality that is unfair but under the control of Governments, leaving room for health improvement through policies for migrant integration. On the other side, interactions between migrant status and individual SES factors may be interpreted as a measure of unfair inequality. Overall, the health of non-EU migrants appears to be negatively affected by living in countries with problems in integration policies. This result holds even when we control for migrant inequalities in SES. The analysis of Model 6 allows assessing the adverse effect of the lack of pro-migrant integration policies on migrant health. To this end, we can calculate the conditional marginal effects at means. The average increase in the probability that a non-EU migrant is sicker than the baseline citizen due to one additional problem with migrant integration policies is 3.8 %.^3^ However, this effect is considerably higher (21 %) when migrant integration policies become highly critical, i.e. the number of problems rises from 0 to 5.

The results for the estimation of multilevel mixed effects ordered logit models for the probability of reporting limitations in daily life are shown in Table 5. Looking at the first model, being a non-EU migrant has a positive effect on the probability of reporting limitations in daily life. However, the odds ratio in Models 2–5 is not significant anymore. Similarly to the results for SAH, Model 6 shows a negative and significant effect of the migrant status on health that could be interpreted as “healthy migrant effect”. The interaction term of the policy variable with the migrant status shows that being a migrant and living in a country with problems of integration increases the odds of reporting health limitations. This result holds for Models 4 and 5, and it is confirmed after controlling for migrant SES (Model 6). However, the effect of migrant integration policies does not hold for the general population, as the estimated odds for the policy variable are below one. The conditional marginal effect at means, i.e. the increase in the probability that a non-EU migrant suffers from LLS as compared to the baseline citizen due to an additional problem in integration policies, is 4.6 %. This effect is much higher (21 %) when migrant integration problems increase from 0 to 5.

Finally, Table 6 shows the results from the estimation of multilevel logit models for the probability of reporting chronic conditions. From the first three models, the non-EU migrant variable has no significant effect on the probability of reporting chronic conditions. Models 4, 5 and 6 show that, once the interaction term with the policy variable is introduced in the estimation, the status of non-EU migrant appears to be associated with lower odds of reporting chronic diseases. Again, this could be due to the “healthy migrant hypothesis”. On the other hand, similarly to the results obtained for the other dependent variables, Models 4–6 show that living in countries where there are problems in integration policies increases the odds of reporting chronic conditions for migrants. Conversely, integration policies do not significantly affect the odds for the rest of the population. The increase in the probability of reporting chronic conditions for the non-EU migrant as compared to the baseline citizen because of an additional problem with migrant integration policies is 4.3 %. Like for SAH and LLS, this difference in probability increases to 21 % when migrant integration problems rise from 0 to 5.

**Fig. 3:**
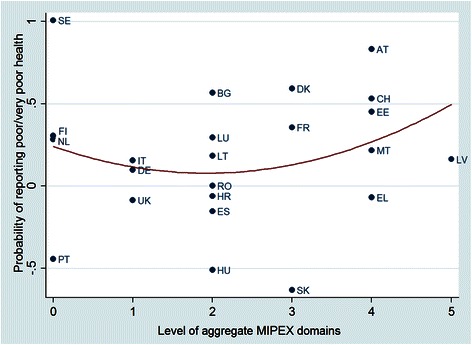
Two-stage logit estimation results – Estimated probability of reporting poor or very poor health for non-EU migrants vs. number of problematic areas of migrant integration policies by country– year: 2012. Legend: AT = Austria, BG = Bulgaria, CH = Switzerland, DE = Germany, DK = Denmark, EE = Estonia, EL = Greece, ES = Spain, FI = Finland, FR = France, HR = Croatia, HU = Hungary, IT = Italy, LT = Lithuania, LU = Luxembourg, LV = Latvia, MT = Malta, NL = The Netherlands, PT = Portugal, RO = Romania, SE = Sweden, SK = Slovak Republic, UK = United Kingdom. Source: Graphical output obtained from Stata v.13 command *mlt2scatter*, using Eurostat [21, 27], OECD [26] data for 2012 and MIPEX [25] data. Results were obtained by running two-stage logit models using Stata v.13 command: *mlt2stage*. In the first step, separate country estimates were obtained by running logit models for the probability of reporting poor or very poor health using only individual level variables and controlling for age, gender, log(income), employment status, marital status and migrant status. In the second step the estimated slopes of the dependent variable for the non-EU citizen status from the first step were plotted against the country-level variable for problems in migrant integration policies

To check the robustness of the findings, we performed a two-step analysis and reported the estimates at country level. In the first step, separate estimates for each country were obtained by running logit models for the probability of reporting poor or very poor health using only individual level variables. In the second step, for each country we plotted the estimated slopes of the dependent variable for non-EU migrants and problems in migrant integration policies. Therefore, it is possible to visualize the interaction effect between the policy variable and the non-EU migrant status (Fig. 3).^4^ The two-step logit analyses for the probability of reporting limitations in daily life and for the probability of reporting chronic conditions confirm the results (Additional files 1 and 2).

For comparison with previous results obtained using the 2007 wave of Eurostat EU-SILC data for a set of 14 European countries, we show that socio-economic health inequalities persist in times of crisis and are driven by the socio-economic status. As expected, individual determinants affect health, as suggested by previous studies [4-9, 16-20]. The self-reported health status of non-EU migrants living in European countries is negatively influenced by the country context in terms of problems in migrant integration. This result holds even when we control for country characteristics and consider more objective measures of the health status, such as limitations in daily life and the presence of chronic conditions. Therefore, living in a country with problems in migrant integration can offset the “healthy migrant effect”.

To conclude, it is worth underlying that this work relies on individual cross-sectional surveys from the EU-SILC dataset. Longitudinal data could not be used because information on some variables was limited in the panel version of the EU-SILC dataset [22]. In order to overcome this limitation and to exploit the whole pseudo-panel of cross-sectional data, further analysis is needed. Finally, further work is planned to include measures of attitudes to migrants from other surveys on citizens’ attitudes and values.

## Conclusions

We examined health inequalities in a set of European countries, allowing for both individual socio-economic determinants of health and country-level characteristics, including migrant integration policies derived from the Migrant Integration Policy Index. This work adds on existing evidence that overall policies for non-European migrant integration can reduce health disparities in times of economic crisis. Our findings reinforce the view that migrant integration policies are needed in order to tackle inequalities in health and ultimately to improve equity in health.

### Declarations

This study is based on Eurostat EU-SILC Cross Sectional Reference year: 2012 [21]. Access to data for scientific purposes has been granted under the current EU regulation. Responsibility for all conclusions drawn from the data lies entirely with the authors.

## Abbreviations

EU, European Union; LLS, Self-reported limiting long-standing illnesses; MIPEX, Migrant integration policy index; SAH, Self-assessed health; SC, Self-reported chronic illness; SES, Socio-economic status.

## Additional files

Additional file 1: Figure S1. Two-stage logit estimation results – Estimated probability of reporting limitations in daily life for non-EU migrants vs. number of problematic areas of migrant integration policies by country– year: 2012.
Legend: AT = Austria, BG = Bulgaria, CH = Switzerland, DE = Germany, DK = Denmark, EE = Estonia, EL = Greece, ES = Spain, FI = Finland, FR = France, HR = Croatia, HU = Hungary, IT = Italy, LT = Lithuania, LU = Luxembourg, LV = Latvia, MT = Malta, NL = The Netherlands, PT = Portugal, RO = Romania, SE = Sweden, SK = Slovak Republic, UK = United Kingdom. Source: Graphical output obtained from Stata v.13 command *mlt2scatter*, using Eurostat [21, 27], OECD [26] data for 2012 and MIPEX [25] data.
Results were obtained by running two-stage logit models using Stata v.13 command: *mlt2stage*. In the first step, separate country estimates were obtained by running logit models for the probability of reporting limitations in daily life using only individual level variables and controlling for age, gender, log(income), employment status, marital status and migrant status. In the second step the estimated slopes of the dependent variable for the non-EU citizen status from the first step were plotted against the country-level variable for problems in migrant integration policies. (PDF 71 kb)

Additional file 2: Figure S2. Two-stage logit estimation results – Estimated probability of reporting chronic conditions for non-EU migrants vs. number of problematic areas of migrant integration policies by country– year: 2012.
Legend: AT = Austria, BG = Bulgaria, CH = Switzerland, DE = Germany, DK = Denmark, EE = Estonia, EL = Greece, ES = Spain, FI = Finland, FR = France, HR = Croatia, HU = Hungary, IT = Italy, LT = Lithuania, LU = Luxembourg, LV = Latvia, MT = Malta, NL = The Netherlands, PT = Portugal, RO = Romania, SE = Sweden, SK = Slovak Republic, UK = United Kingdom. Source: Graphical output obtained from Stata v.13 command *mlt2scatter*, using Eurostat [21, 27], OECD [26] data for 2012 and MIPEX [25] data.
Results were obtained by running two-stage logit models using Stata v.13 command: *mlt2stage.* In the first step, separate country estimates were obtained by running logit models for the probability of reporting chronic conditions using only individual level variables and controlling for age, gender, log(income), employment status, marital status and migrant status. In the second step the estimated slopes of the dependent variable for the non-EU citizen status from the first step were plotted against the country-level variable for problems in migrant integration policies. (PDF 69 kb)

## Footnotes

1 We excluded Iceland from the analysis, because no MIPEX data were available for this country. Moreover, observations for Poland and Norway were excluded from the initial sample due to the presence of several missing data on citizenship. Similarly, Cyprus, Czech republic and Slovenia were excluded from the analysis because of data limitations.

2 In this case, we used the interaction term between the migrant status and a set of categorical variables for low education, low income (lowest tertile of individual income), marital status (not married, single or separated, widow), and employment status (unemployed, self-employed, housework). We also estimated models including the six separate dimensions of the MIPEX index (Table 3). This allows for the estimation of the contribution of migrant integration policies in specific areas, namely the acquisition of nationality, political participation, labor market mobility, anti discrimination, long-term residence, and family reunion. However, the MIPEX domains were highly correlated and the estimated odds were all significant and very close to one. Therefore, this specification did not seem to convey any clear message useful for policy purposes. We decided not to report these results in the paper.

3 Details on marginal effects calculated using the Delta method are available upon request.

4 The graphs shown are the scatter plot output from Stata v.13 routine *mlt2scatter* for the probability of reporting poor health status. This has been used together with the routine *mlt2stage* in order to produce two-stage plots of the estimated country-level regression coefficients of the individual lower-level variable for migrant status over the country higher-level variable measuring problems in integration policies.
