# Supplementary figures and images for: Migrant integration policies and health inequalities in Europe
Source: BMC Public Health. 2016 Jun 1;16:463. doi: 10.1186/s12889-016-3095-9 (PMC4888480; doi:10.1186/s12889-016-3095-9)

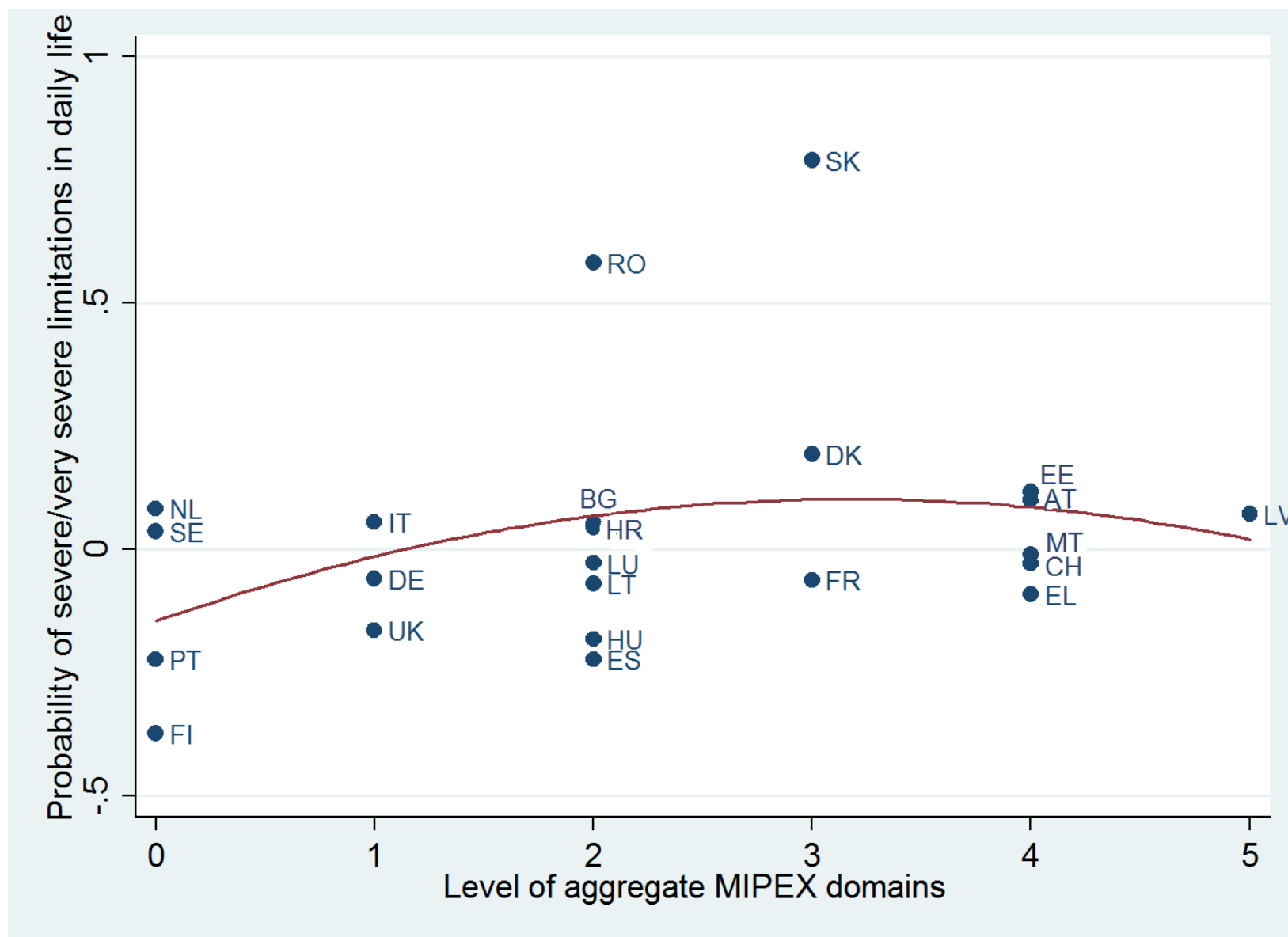

Supplement: Supplementary file 1 — Two-stage logit estimation results – Estimated probability of reporting limitations in daily life for non-EU migrants vs. number of problematic areas of migrant integration policies by country– year: 2012. Legend: AT = Austria, BG = Bulgaria, CH = Switzerland, DE = Germany, DK = Denmark, EE = Estonia, EL = Greece, ES = Spain, FI = Finland, FR = France, HR = Croatia, HU = Hungary, IT = Italy, LT = Lithuania, LU = Luxembourg, LV = Latvia, MT = Malta, NL = The Netherlands, PT = Portugal, RO = Romania, SE = Sweden, SK = Slovak Republic, UK = United Kingdom. Source: Graphical output obtained from Stata v.13 command mlt2scatter, using Eurostat [21, 27], OECD [26] data for 2012 and MIPEX [25] data. Results were obtained by running two-stage logit models using Stata v.13 command: mlt2stage. In the first step, separate country estimates were obtained by running logit models for the probability of reporting limitations in daily life using only individual level variables and controlling for age, gender, log(income), employment status, marital status and migrant status. In the second step the estimated slopes of the dependent variable for the non-EU citizen status from the first step were plotted against the country-level variable for problems in migrant integration policies. (PDF 71 kb) [file 12889_2016_3095_MOESM1_ESM.pdf]

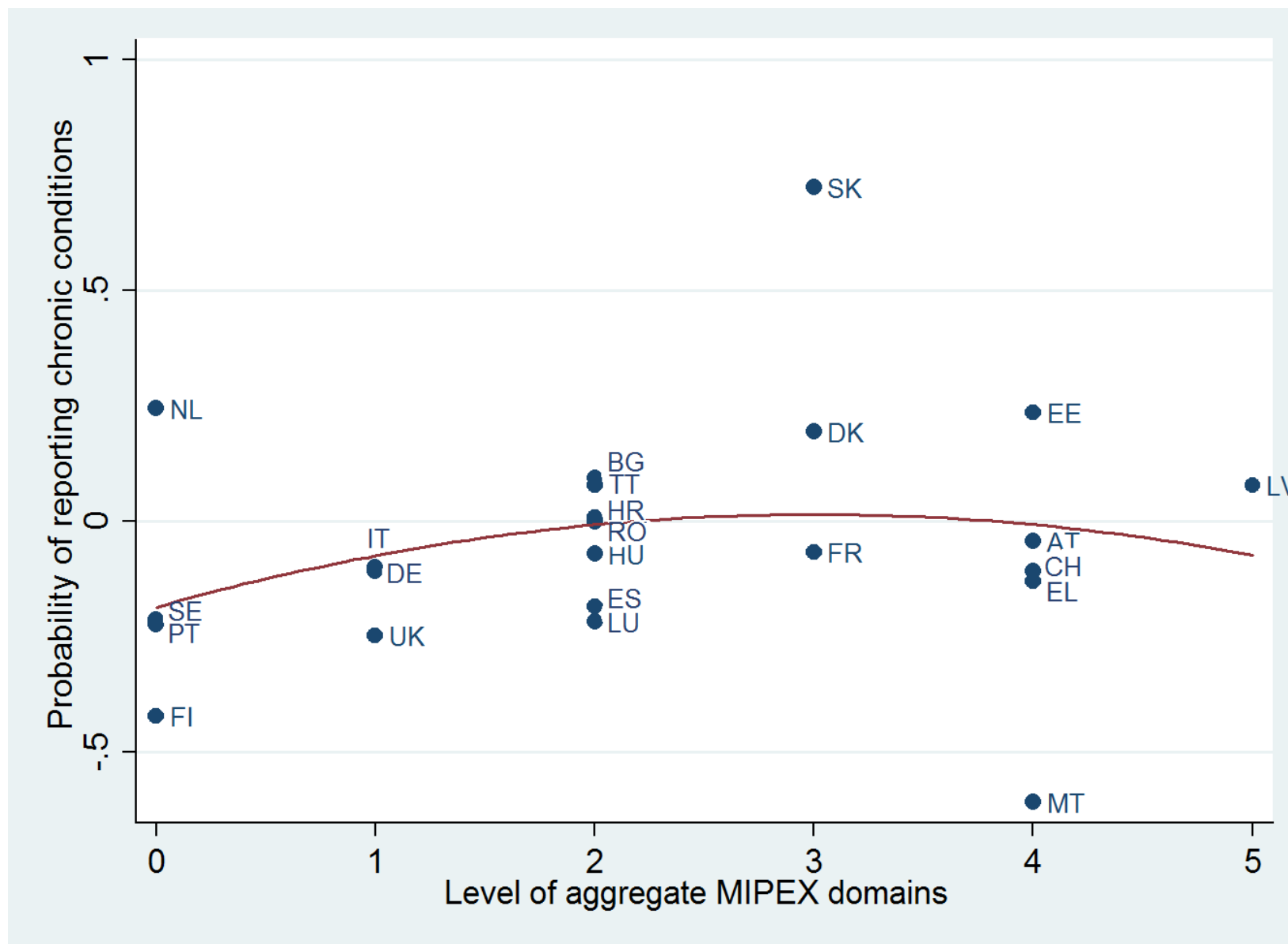

Supplement: Supplementary file 2 — Two-stage logit estimation results – Estimated probability of reporting chronic conditions for non-EU migrants vs. number of problematic areas of migrant integration policies by country– year: 2012. Legend: AT = Austria, BG = Bulgaria, CH = Switzerland, DE = Germany, DK = Denmark, EE = Estonia, EL = Greece, ES = Spain, FI = Finland, FR = France, HR = Croatia, HU = Hungary, IT = Italy, LT = Lithuania, LU = Luxembourg, LV = Latvia, MT = Malta, NL = The Netherlands, PT = Portugal, RO = Romania, SE = Sweden, SK = Slovak Republic, UK = United Kingdom. Source: Graphical output obtained from Stata v.13 command mlt2scatter, using Eurostat [21, 27], OECD [26] data for 2012 and MIPEX [25] data. Results were obtained by running two-stage logit models using Stata v.13 command: mlt2stage. In the first step, separate country estimates were obtained by running logit models for the probability of reporting chronic conditions using only individual level variables and controlling for age, gender, log(income), employment status, marital status and migrant status. In the second step the estimated slopes of the dependent variable for the non-EU citizen status from the first step were plotted against the country-level variable for problems in migrant integration policies. (PDF 69 kb) [file 12889_2016_3095_MOESM2_ESM.pdf]
